# Extracorporeal Membrane Oxygenation in the Management of Takotsubo Cardiomyopathy Secondary to Mitral Valve Replacement Surgery: A Case Report

**DOI:** 10.7759/cureus.71258

**Published:** 2024-10-11

**Authors:** Syed Mohammed, Saptarshi Maitra

**Affiliations:** 1 Critical Care Medicine, Madinah Cardiac Center, Madinah, SAU; 2 Critical Care Medicine, Peterborough City Hospital, Peterborough, GBR

**Keywords:** cardiogenic shock, mitral valve replacement, postoperative complications, stress-induced cardiomyopathy, takotsubo cardiomyopathy (tc), venoarterial extracorporeal membrane oxygenation (va-ecmo)

## Abstract

Unlike classical myocardial infarction, acute emotional or physical stress can trigger distinct but transient wall motion abnormalities with patent coronary arteries. Takotsubo cardiomyopathy (TC) classically presents as systolic and diastolic left ventricular (LV) dysfunction, which is widely accepted to be secondary to catecholamine-induced microvascular dysfunction.

Our case report describes a 26-year-old female undergoing mitral valve replacement (MVR) surgery for infective endocarditis and postoperatively developing TC. Severe hemodynamic instability with low mean arterial pressure necessitated emergency intubation and ventilation. Bedside formal echocardiography reported mid and apical segment akinesia with significant left ventricular systolic dysfunction.

This case identifies a potential cause of sudden-onset heart failure (HF) in patients with critical illness. The early recognition and appropriate management of TC are highlighted in this article, particularly in high-risk patients undergoing cardiac surgery. In recent years, extracorporeal membrane oxygenation (ECMO) has presented a unique opportunity to mechanically manipulate pulmonary vascular pressure. TC is a unique and uncommon cause of sudden-onset heart failure in patients with critical illnesses. This article illustrates the importance of the early recognition and management of TC with ECMO.

Considering the rarity of TC, randomized controlled trials are yet to establish optimal management strategies for specific etiology. Further research and case series are vital to enhance our understanding of the vital use of ECMO in the treatment of takotsubo cardiomyopathy secondary to MVR.

## Introduction

Takotsubo cardiomyopathy (TC), also known as stress-induced cardiomyopathy or apical ballooning syndrome, is a transient systolic and diastolic left ventricular (LV) dysfunction characterized by distinct wall motion abnormalities with normal coronary artery findings. First described in Japanese literature in 1990, the term "takotsubo" is derived from the shape of a Japanese ceramic pot used for catching octopus, reflecting the unique pattern of the left ventricle with apical ballooning and a hyperkinetic base. This condition is often triggered by acute emotional or physical stress and predominantly affects postmenopausal females, typically resolving within 3-6 months. The most widely accepted etiology is catecholamine-induced microvascular dysfunction [1].

Patients undergoing cardiac surgery are particularly vulnerable to cardiac toxicity induced by catecholamines due to direct cardiac stimulation during the procedure. Cardiogenic shock is, however, not uncommon postoperatively, but like in our case described below, it is vital that the underlying cause is identified and managed accordingly [1,2].

We report the case of a 26-year-old female with TC post-mitral valve surgery for infective endocarditis, managed with extracorporeal membrane oxygenation (ECMO).

## Case presentation

A 26-year-old female with mitral valve endocarditis was admitted for mitral valve replacement surgery. She had previously undergone a five-week course of antibiotics for suspected infective endocarditis complicated by splenic infarction. The causative organisms were *Streptococcus viridans* and *Brucella melitensis*, identified in postoperative cardiac tissue microscopy and culture. Preoperative echocardiography confirmed normal left ventricular ejection fraction (LVEF) with large vegetation on both mitral valve leaflets.

Anesthesia induction and maintenance were performed as per standard protocol, with routine cardiac aesthetic monitoring. A median sternotomy was performed, and the patient was placed on standard cardiopulmonary bypass with cardioplegic cardiac arrest. The mitral valve was accessed through a left atrial incision. After excising the infected leaflets, the native mitral valve was replaced with a 31 mm mosaic bioprosthesis, preserving the posterior mitral leaflet and annulo-ventricular continuity. Intraoperative transesophageal echocardiography revealed good mitral prosthetic function, no paravalvular leak, moderate global hypokinesia with an ejection fraction (EF) of approximately 40%, regional left ventricular contractions, and normal right ventricular function. Cardiopulmonary bypass time was 83 minutes, and aortic cross-clamp time was 58 minutes.

Postoperatively (postoperative day {POD} 1), the patient was successfully weaned off cardiopulmonary bypass and transferred to the Cardiac Surgery Intensive Care Unit (CICU) with high inotropic support. On arrival, she was intubated and ventilated. The arterial blood pressure (BP) was reported at 90/47 mmHg with 0.1 mcg/kg/minute noradrenaline and 0.07 mcg/kg/minute epinephrine support. Sedation was maintained with fentanyl and midazolam. Due to a drop in hemoglobin, two units of packed red blood cells (pRBC) were transfused. A 12-lead ECG revealed sinus tachycardia with a heart rate of approximately 130/minute and T-wave inversion in the precordial leads.

**Figure 1:**
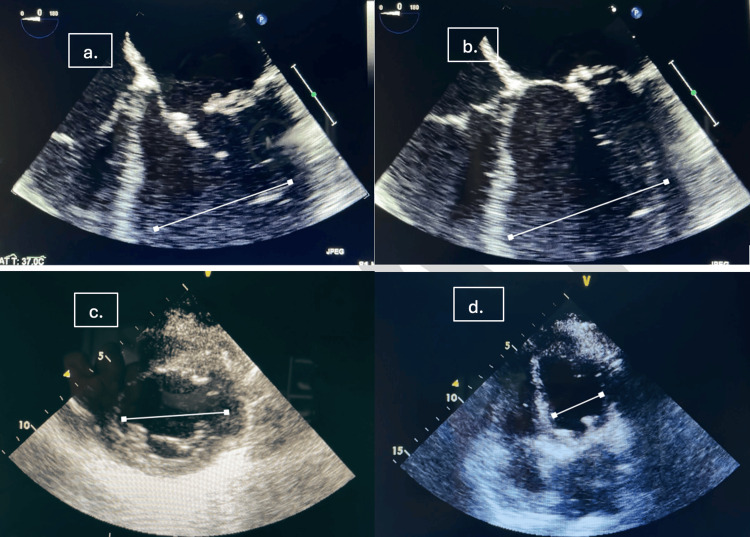
Serial echocardiography to illustrate the various stages of recovery over the course of admission. (a and b) The initial imaging performed on postoperative day (POD) 4, identifying mid and apical segment akinesia with contracting basal segments. Normal LV size with a reduced ejection fraction of 20%. (c) Echocardiography performed on postoperative day 9 on ECMO, identifying improved mid and apical contractility and an improved ejection fraction of 45%. (d) POD 15 and normal LV size with a moderately reduced ejection fraction of 35%. LV, left ventricular; ECMO, extracorporeal membrane oxygenation

Considering the high inotropic support four days postoperatively (arterial BP was recorded at 92/51 mmHg on 0.7 mcg/kg/minute noradrenaline, and vasopressin was escalated to 3.0 mL/hour), a transthoracic echocardiogram was performed, revealing mid and apical segment akinesia with contracting basal segments and an LVEF of 20% (as shown in Figure 1a, 1b). The ECG was unchanged with persistently elevated brain natriuretic peptide (BNP), while troponin and creatine kinase-myocardial band (CK-MB) were not significantly elevated. Subsequent invasive coronary angiography revealed patent coronary arteries. Arterial blood gas identified elevated lactate (4.6-7.6), base excess of -6.8, over a 24-hour period. Given her persistent hemodynamic instability despite pharmacotherapy, elevated lactate, and persistently raised BNP (Society for Cardiovascular Angiography & Interventions {SCAI} Shock Stage D), venoarterial extracorporeal membrane oxygenation (VA-ECMO) was considered appropriate and implemented. Right femoral catheterization provided adequate access. Considering the increased afterload associated with VA-ECMO, LV offloading strategies such as fluid optimization and intra-aortic balloon pump (IABP) were utilized. Post-ECMO insertion, the patient was paralyzed with cisatracurium (2 mcg/kg/minute) and started on broad-spectrum antibiotics (meropenem and vancomycin). Lactate levels recorded after VA-ECMO insertion and 24-hour utilization were in the range of 1.6-2.4.

On the ninth POD, repeat echocardiography revealed improved LVEF, from 20% previously to 45% (as shown in Figure 1c). She was successfully weaned off ECMO, followed by extubation the next day. The follow-up day 15 echocardiography reported a bioprosthetic mitral valve with an acceptable gradient and an impaired LV ejection fraction of 35% (as shown in Figure 1d). She was advised to undergo routine screening to monitor her ejection fraction annually. The series of echocardiographs shown in Figure 1 reflect the stages of evolution of takotsubo cardiomyopathy postoperatively.

## Discussion

Takotsubo cardiomyopathy (TC), sometimes referred to as stress-induced cardiomyopathy, is a sudden, usually reversible syndrome that causes transient heart failure (HF). TC presents clinically similarly to acute coronary syndrome (ACS), which initially makes the diagnosis and management of the condition difficult. A thorough assessment is necessary in order to pinpoint its defining characteristics. In this case report, we want to illustrate the need to consider extracorporeal membrane oxygenation (ECMO) in patients with TC post-cardiac surgery.

In patients with TC, plasma catecholamine concentrations are significantly elevated compared to those with other cardiac conditions, such as acute myocardial infarction. The prevention of psychological, physical, and pharmacological sympathomimesis through aggressive pain management, adequate sedation, and non-pharmacological cardiovascular support reduces the probability of the development of TC following surgery. A study on 2078 patients suffering from TC identified cardiogenic shock as complicating the in-hospital course of ≈10% of patients with TC [1-3].

In our case, the hemodynamic instability is classically seen in acute heart failure. Due to the limitation of the availability of drugs (e.g., levosimendan and milrinone), a plan of early initiation of ECMO with appropriate LV unloading was made in the best interest and aims to illustrate the utility of ECMO in TC secondary to mitral valve replacement surgery. Further escalation of inotropic support with levosimendan and milrinone has been recommended. The Randomised stUdy on Safety and effectivenesS of Levosimendan in patients with left ventricular failure due to an Acute myocardial iNfarct (RUSSLAN), one of the largest placebo-controlled studies studying the use of levosimendan in acute heart failure, showed a decreased incidence of worsening HF and improvements in short- and long-term mortality with the use of levosimendan in patients with EF of ≤30% [4].

**Figure 2:**
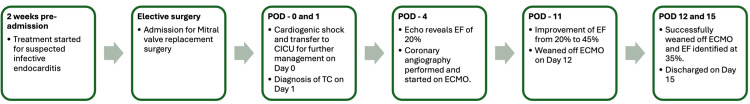
Illustrative summary of clinical course in 26-year-old female admitted for mitral valve replacement and the eventual management of takotsubo cardiomyopathy (TC). EF, ejection fraction; POD, postoperative day; CICU, Cardiac Surgery Intensive Care Unit; ECMO, extracorporeal membrane oxygenation

In recent years, ECMO has presented a unique opportunity to mechanically manipulate pulmonary vascular pressure. TC is a unique and uncommon cause of sudden-onset heart failure in patients with critical illnesses. This article illustrates the importance of the early recognition and management of TC with ECMO. As shown in Figure 2, to limit the use of inotropic support during her course of recovery, left ventricular assist devices such as intra-aortic balloon pumps have been recommended.

During ECMO, venous blood is drawn out to achieve complete oxygenation and then injected into the artery at an appropriate flow rate to ensure the patient's acceptable oxygenation and mean arterial pressure. Additionally, in cardiogenic shock, the left ventricle typically lacks the preload and contractile reserve necessary to compensate for the elevated afterload associated with VA-ECMO, therefore elevating myocardial oxygen demand in an already compromised ventricle. The intra-aortic balloon pump (IABP) is the most commonly utilized adjunctive mechanical circulatory support device for left ventricular unloading. It is nestled in a conventional position within the descending aorta, where deflation during systole reduces afterload during left ventricular ejection and facilitates forward flow through the aortic valve, while inflating during diastole enhances coronary blood flow. The use of IABP is under evaluation, requiring balancing the risk of worsening heart failure with the risk of major hemorrhage associated with a medical prosthetic device [5,6].

Patients with a cardiac index of <2 L/minute/mm^2^, systolic blood pressure of <90 mmHg, and lactic acidosis despite inotropic support and IABP should be considered for ECMO. ECMO resuscitation can serve as a bridge to heart transplantation or a ventricular-assisted device in cases of irreversible cardiac failure [7].

ECMO resulted in a rapid improvement in serological parameters, a full reversal of cardiac failure, and improvement in prognosis in this case when all conservative pharmacological therapy and fluid management interventions failed to restore cardiac function.

## Conclusions

ECMO provides appropriate reinjection of oxygenated blood to ensure appropriate mean arterial pressure and systemic oxygenation. Patients with significant wall motion abnormalities and takotsubo cardiomyopathy post-cardiac surgery benefit significantly from early extracorporeal membrane oxygenation support, as evidenced in this case report.
